# Supplementary material for: A Genetic Study of Spillovers in the Bean Common Mosaic Subgroup of Potyviruses
Source: Viruses. 2024 Aug 23;16(9):1351. doi: 10.3390/v16091351 (PMC11436247; doi:10.3390/v16091351)
Supplement: Supplementary file 1 [file viruses-16-01351-s001.zip › viruses-3153996-supplementary.pdf]

Supplementary Data Table 1. Accession Numbers, Hosts and Provenances

Virus	C v N-C	Virus	AccCode	Host	Host-fam	Country
		BCMNV	KX302007	Phaseolus vulgaris	Fabaceae	East Timor
		BCMNV	KY659304	P. vulgaris	Fabaceae	USA
		BCMNV	MG640393	P. leptostachyus	Fabaceae	Mexico
		BCMNV	MG640394	P.vulgaris	Fabaceae	Mexico
		BCMNV	MG640395	P.vulgaris	Fabaceae	Mexico
		BCMNV	MG640396	P.vulgaris	Fabaceae	Mexico
		BCMNV	MG640397	P.vulgaris	Fabaceae	Mexico
		BCMNV	MG640398	P.vulgaris	Fabaceae	Mexico
		BCMNV	MG640399	P.vulgaris	Fabaceae	Mexico
		BCMNV	MH169568	P.vulgaris	Fabaceae	Kenya
		BCMNV	MK069982	P.vulgaris	Fabaceae	Mexico
		BCMNV	MK069983	P.vulgaris	Fabaceae	Mexico
		BCMNV	MK069984	P.vulgaris	Fabaceae	Mexico
		BCMNV	MW731696	Lablab purpureus	Fabaceae	Bangladesh
		BCMNV	MW731697	L. purpureus	Fabaceae	Bangladesh
		BCMNV	MW731698	L. purpureus	Fabaceae	Bangladesh
	crop	BCMNV	MW731699	L. purpureus	Fabaceae	Bangladesh
		SbMV	FJ640956	wild soybean	Fabaceae	Korea
		SbMV	KM979229	G. max	Fabaceae	India
		EAPV	KP114137	Passiflora spp.	Passifloraceae	Taiwan
		SbMV	KP710872	G. max	Fabaceae	China
		CpAbMV	KT726938	Vigna unguiculata	Fabaceae	Uganda
		EAPV	KY614052	Passiflora spp.	Passifloraceae	Taiwan
		UrMV	LC477217	P.edulis	Passifloraceae	Japan
		CpAbMV	MF179118	V. unguiculata	Fabaceae	Kenya
		SbMV	MH428831	G. max	Fabaceae	Korea
		WVMV	MN514947	Wisteria spp.	Fabaceae	Iran
		PasCLV	MT263075	Passiflora spp.	Passifloraceae	Israel
		PaMNV	MW187786	Polygonatum kingianum	Asparagaceae	China
		FritVY	NC_010954	Fritillaria thunbergii	Liliaceae	China
		SafLV	NC_036802	Crocus sativus	Iridaceae	Iran
BCMNV	outgroup	PasCLV	OL584353	P. edulis	Passifloraceae	nd
		PstV	AY968604	Peanut	Fabaceae	China
		BCMV	EU761198	Macroptilium atropurpureum	Fabaceae	Australia
		BCMV	KF114860	P. vulgaris	Fabaceae	India
		BCMV	KF439722	Arachis hypogaea	Fabaceae	China
		BCMV	KJ508092	G. max	Fabaceae	Korea
		BCMV	KJ807799	G. max	Fabaceae	China
		BCMV	KJ807801	G. max	Fabaceae	China
		BCMV	KJ807812	G. max	Fabaceae	China
BCMV	crop	BCMV	KJ807813	G. max	Fabaceae	China

		BCMV	KJ807815	G. max	Fabaceae	China
		BCMV	KJ807820	G. max	Fabaceae	China
		BCMV	KM023744	P. vulgaris	Fabaceae	USA
		BCMV	KM051425	P. vulgaris	Fabaceae	China
		BCMV	KM051427	G. max	Fabaceae	China
		BCMV	KM051428	G. max	Fabaceae	China
		BCMV	KM051430	G. max	Fabaceae	China
		BCMV	KM076650	Cudrania tricuspidata	Moraceae	Korea
		BCMV	KT175568	P. vulgaris	Fabaceae	USA
		BCMV	KU896809	P. vulgaris	Fabaceae	USA
		BCMV	LC582403	V. radiata	Fabaceae	China
		BCMV	MF405191	P. vulgaris	Fabaceae	Tanzania
		BCMV	MG640401	P. vulgaris	Fabaceae	Mexico
		BCMV	MG640402	P. acutifolius	Fabaceae	Mexico
		BCMV	MG640404	P. vulgaris	Fabaceae	Mexico
		BCMV	MG640406	P. vulgaris	Fabaceae	Mexico
		BCMV	MG640408	P. vulgaris	Fabaceae	Mexico
		BCMV	MG640409	P. vulgaris	Fabaceae	Mexico
		BCMV	MG640410	P. vulgaris	Fabaceae	Mexico
		BCMV	MG640411	P. vulgaris	Fabaceae	Mexico
		BCMV	MG640413	P. vulgaris	Fabaceae	Mexico
		BCMV	MH024839	P. vulgaris	Fabaceae	USA
		BCMV	MH024841	P. vulgaris	Fabaceae	USA
		BCMV	MH220846	P. vulgaris	Fabaceae	Australia
		BCMV	MH220847	P. vulgaris	Fabaceae	Australia
		BCMV	MH568695	Sesamum indicum	Pedaliaceae	China
		BCMV	MH744999	Vigna unguiculata	Fabaceae	India
		BCMV	MK069986	P. vulgaris	Fabaceae	Mexico
		BCMV	MN399727	legume	Fabaceae	Germany
		BCMV	MN786956	Arachis hypogeae	Fabaceae	China
		BCMV	MN987558	P. vulgaris	Fabaceae	Zambia
		PstV	MZ442684	A. hypogeae	Fabaceae	Turkey
		BCMV	MZ670770	Nandina domestica	Berberidaceae	China
		BCMV	OL871237	Pachyrhizus erosus	Fabaceae	China
		PstV	U05771	A. hypogea	Fabaceae	USA
		PstV	U34972	A. hypogea	Fabaceae	USA
		SbMV	FJ640956	wild soybean	Fabaceae	Korea
		BCMV	KM051429	G. max	Fabaceae	China
		SbMV	KM979229	G. max	Fabaceae	India
		EAPV	KP114137	Passiflora spp.	Passifloraceae	Taiwan
		SbMV	KP710872	G. max	Fabaceae	China
		UrMV	LC477217	P.edulis	Passifloraceae	Japan
	outgroup	SbMV	MH428831	G. max	Fabaceae	Korea

		WVMV	MN514947	Wisteria spp.	Fabaceae	Iran
		PaMNV	MW187786	P. kingianum	Asparagaceae	China
		YBMV	MZ018226	Canavalia ensiformis	Fabaceae	Brazil
		WVMV	NC_007216	Wisteria spp.	Fabaceae	China
		TelMV	NC_009742	Telosma cordata	Apocynaceae	Vietnam
		PaMNV	NC_043532	Daiswa polyphylla	Melanthiaceae	China
		PaMNV	OM128429	Paris polyphylla	Melanthiaceae	China
		EAPV	OM621805	P. edulis	Passifloraceae	Korea
		CpAbMV	KT726938	V. unguiculata	Fabaceae	Uganda
		CpAbMV	MF179118	V. unguiculata	Fabaceae	Kenya
		CpAbMV	MF179120	V. unguiculata	Fabaceae	Kenya
		CpAbMV	MW582799	V. unguiculata	Fabaceae	Nigeria
		CpAbMV	NC_004013	V. unguiculata	Fabaceae	Zimbabwe
		CpAbMV	OK558658	V. unguiculata	Fabaceae	Nigeria
		CpAbMV	OK558659	V. unguiculata	Fabaceae	Nigeria
	crop	CpAbMV	OK558660	V. unguiculata	Fabaceae	Nigeria
		SbMV	FJ640956	wild soybean	Fabaceae	Korea
		SbMV	KM979229	G. max	Fabaceae	India
		EAPV	KP114137	Passiflora spp.	Passifloraceae	Taiwan
		SbMV	KP710872	G. max	Fabaceae	China
		EAPV	KY614052	Passiflora spp.	Passifloraceae	Taiwan
		UrMV	LC477217	P. edulis	Passifloraceae	Japan
		WVMV	MN514947	Wisteria spp.	Fabaceae	Iran
		PaCV	MT263075	P. edulis	Passifloraceae	Israel
		PMNV	MW187786	P. kingianum	Asparagaceae	China
		CpAbMV	MW534369	P. vulgaris	Fabaceae	Zambia
		BCMNV	MW731696	L. purpureus	Fabaceae	Bangladesh
		WVMV	NC_007216	Wisteria spp.	Fabaceae	China
		PaMNV	NC_043532	Daiswa polyphylla	Melanthiaceae	China
		PaCV	OL584353	Passiflora sp	Passifloraceae	nd
CpAbMV	outgroup	PaMNV	OM128429	Paris polyphylla	Melanthiaceae	China
		DashMV	KT026108	Colocasia esculenta	Araceae	India
		VanMV	KX505964	Vanilla x tahitensis	Orchidaceae	Cook Islands
		DashMV	KY242358	C. esculenta	Araceae	USA
		DashMV	KY242359	C. esculenta	Araceae	USA
		DashMV	MG602228	C. esculenta	Araceae	Ethiopia
		DashMV	MG602229	C. esculenta	Araceae	Ethiopia
		DashMV	MG602230	C. esculenta	Araceae	Ethiopia
		DashMV	MG602231	Xanthosoma sp.	Araceae	Ethiopia
		DashMV	MG602235	Xanthosoma sp	Araceae	Uganda
		DashMV	MZ420678	Typhonium giganteum	Araceae	China
	crop	DashMV	ON086743	C. esculenta	Araceae	Papua-NG
DashMV	outgroup	PWV	AB761400	P. edulis	Passifloraceae	Australia

		HarMV	KJ152152	Hardenbergia comptoniana	Fabaceae	Australia
		HarMV	KJ152153	Lupinus cosentinii	Fabaceae	Australia
		HarMV	KJ152154	H. comptoniana	Fabaceae	Australia
		HarMV	KJ152156	H. comptoniana	Fabaceae	Australia
		PWV	KX577780	P. caerulea	Passifloraceae	Australia
		PfVY	LC466655	P. edulis	Passifloraceae	Japan
		WVMV	MN514947	Wisteria spp.	Fabaceae	Iran
		PaMNV	MW187786	Polygonatum kingianum	Asparagaceae	China
		PaVY	MZ190341	G. max	Fabaceae	Brazil
		WVMV	NC_007216	Wisteria spp.	Fabaceae	China
		TelMV	NC_009742	Telosma cordata	Apocynaceae	Vietnam
		HarMV	NC_015394	H. comptoniana	Fabaceae	Australia
		BSVA	NC_019415	Diuris sp.	Orchidaceae	Australia
		PaMNV	NC_043532	Daiswa polyphylla	Melanthiaceae	China
		EAPV	AB246773	Passiflora spp.	Passifloraceae	Japan
		EAPV	AB690448	Passiflora spp.	Passifloraceae	Japan
		EAPV	AB690449	Passiflora spp.	Passifloraceae	Japan
		EAPV	AB690450	Passiflora spp.	Passifloraceae	Japan
		EAPV	AB690451	Passiflora spp.	Passifloraceae	Japan
		EAPV	AB690452	Passiflora spp.	Passifloraceae	Japan
		EAPV	AB690453	Passiflora spp.	Passifloraceae	Japan
		EAPV	AB690454	Passiflora spp.	Passifloraceae	Japan
		EAPV	AB690455	Passiflora spp.	Passifloraceae	Japan
		EAPV	KP114136	Passiflora spp.	Passifloraceae	Taiwan
		EAPV	KP114137	Passiflora spp.	Passifloraceae	Taiwan
		EAPV	KY614052	Passiflora spp.	Passifloraceae	Taiwan
		EAPV	LC038070	P. edulis	Passifloraceae	Japan
		EAPV	LC038071	P. edulis	Passifloraceae	Japan
		EAPV	LC038072	P. edulis	Passifloraceae	Japan
		EAPV	LC038073	P. edulis	Passifloraceae	Japan
		EAPV	LC038077	P. edulis	Passifloraceae	Japan
		EAPV	LC038078	P. edulis	Passifloraceae	Japan
		EAPV	LC038079	P. edulis	Passifloraceae	Japan
		EAPV	LC038080	P. edulis	Passifloraceae	Japan
		EAPV	LC038081	P. edulis	Passifloraceae	Japan
		EAPV	LC038082	P. edulis	Passifloraceae	Japan
		EAPV	LC325839	Passiflora spp.	Passifloraceae	Japan
		EAPV	LC656468	P. edulis	Passifloraceae	Korea
		EAPV	MT450870	Passiflora spp.	Passifloraceae	Vietnam
		EAPV	NC_007728	Passiflora spp.	Passifloraceae	Japan
		EAPV	OM621805	P. edulis	Passifloraceae	Korea
	crop	EAPV	ON641738	P. edulis	Passifloraceae	China
EAPV	outgroup	SbMV	FJ640956	wild soybean	Fabaceae	Korea

		SbMV	KM979229	G. max	Fabaceae	India
		SbMV	KP710872	G. max	Fabaceae	China
		SbMV	LC037232	Uraria crinita	Fabaceae	Taiwan
		UrMV	LC477217	P. edulis	Passifloraceae	Japan
		WVMV	LC729727	Canavalia ensiformis	Fabaceae	Korea
		SbMV	MH428831	G. max	Fabaceae	Korea
		WVMV	MN514947	Wisteria spp.	Fabaceae	Iran
		WVMV	MT603851	G. max	Fabaceae	Korea
		PMNV	MW187786	Polygonatum kingianum	Asparagaceae	China
		WVMV	NC_007216	Wisteria spp.	Fabaceae	China
		TelMV	NC_009742	Telosma cordata	Apocynaceae	Vietnam
		FVY	NC_010954	Fritillaria thunbergii	Liliaceae	China
		PaMNV	NC_043532	Daiswa polyphylla	Melanthiaceae	China
		PaMNV	OM128429	Paris polyphylla	Melanthiaceae	China
		PeMoV	KF977830	G. max	Fabaceae	Korea
		PeMoV	MH270528	A. hypogeae	Fabaceae	China
		PeMoV	MK396065	A. pintoi	Fabaceae	Brazil
		PeMoV	MT603815	G. max	Fabaceae	Korea
		PeMoV	MT603816	G. max	Fabaceae	Korea
		PeMoV	MT603817	G. max	Fabaceae	Korea
		PeMoV	MT603818	G. max	Fabaceae	Korea
		PeMoV	MT603819	G. max	Fabaceae	Korea
		PeMoV	MT603852	G. max	Fabaceae	Korea
		PeMoV	MT790744	G. max	Fabaceae	China
		PeMoV	MW464637	G. max	Fabaceae	Iran
		PeMoV	MW534370	P. vulgaris	Fabaceae	Zambia
		PeMoV	MZ442685	A. hypogeae	Fabaceae	Turkey
		PeMoV	MZ442686	A. hypogeae	Fabaceae	Turkey
	crop	PeMoV	MZ442687	A. hypogeae	Fabaceae	Turkey
		BaRMV	FJ640956	Basella rubra	Basellaceae	Taiwan
		FreMV	KM979229	Lachenalia sp.	Hyacinthaceae	South Africa
		FreMV	KP710872	Lachenalia sp.	Hyacinthaceae	Korea
		Polygonatum kingianum virus 4	LC037232	Polygonatum kingianum	Asparagaceae	China
		Polygonatum kingianum virus 3	LC477217	P. kingianum	Asparagaceae	China
		BaRMV	LC729727	Anredera cordifolia	Basellaceae	China
		ABMV	MH428831	Achyranthes bidentata	Amaranthaceae	China
		BtMV	MN514947	Beta vulgaris	Amaranthaceae	nd
		BaRMV	MT603851	Anredera cordifolia	Basellaceae	Taiwan
		PlFBV	MW187786	Pleione humilis	Orchidaceae	Czechia
		BFBV	NC_007216	Begonia bowerae cv. Tiger	Begoniaceae	China
		BtMV	NC_009742	B. vulgaris	Amaranthaceae	China
PeMoV	outgroup	BaRMV	NC_010954	Basella alba	Basellaceae	USA

		BFBV	NC_043532	Begonia sp.	Begoniaceae	Germany
		PMaV1	OM128429	Polygonatum cyrtonema Hua	Asparagaceae	China
	crop	SbMV	MH428831	G. max	Fabaceae	Korea
		SbMV	FJ640956	wild soybean	Fabaceae	Korea
		SbMV	KM979229	G. max	Fabaceae	India
		EAPV	KP114137	Passiflora spp.	Passifloraceae	Taiwan
		SbMV	KP710872	G. max	Fabaceae	China
		EAPV	KY614052	Passiflora spp.	Passifloraceae	Taiwan
		SbMV	LC037232	Uraria crinita	Fabaceae	Taiwan
		UrMV	LC477217	P. edulis	Passifloraceae	Japan
		WVMV	MN514947	Wisteria spp.	Fabaceae	Iran
		PaMNV	MW187786	Polygonatum kingianum	Asparagaceae	China
		WVMV	NC_007216	Wisteria spp.	Fabaceae	China
		TelMV	NC_009742	Telosma cordata	Apocynaceae	Vietnam
		FVY	NC_010954	Fritillaria thunbergii	Liliaceae	China
		SaLV	NC_036802	Crocus sativus	Iridaceae	Iran
		PaMNV	NC_043532	Daiswa polyphylla	Melanthiaceae	China
SbMV	outgroup	PaMNV	OM128429	Paris polyphylla	Melanthiaceae	China
		ZYMV	AJ316229	Benincasa hispida	Cucurbitaceae	China
		ZYMV	AY188994	Cucurbita pepo	Cucurbitaceae	Israel
		ZYMV	DQ124239	C. pepo	Cucurbitaceae	Slovakia
		ZYMV	EF062582	C. pepo	Cucurbitaceae	Israel
		ZYMV	EF062583	C. pepo	Cucurbitaceae	Israel
		ZYMV	JN183062	C. pepo	Cucurbitaceae	Iran
		ZYMV	JQ716413	C. pepo	Cucurbitaceae	USA
		ZYMV	KC665627	C. pepo	Cucurbitaceae	USA
		ZYMV	KC665628	C. pepo	Cucurbitaceae	USA
		ZYMV	KC665629	C. pepo	Cucurbitaceae	USA
		ZYMV	KC665630	C. pepo	Cucurbitaceae	USA
		ZYMV	KC665631	C. pepo	Cucurbitaceae	USA
		ZYMV	KC665632	C. pepo	Cucurbitaceae	USA
		ZYMV	KC665633	C. pepo	Cucurbitaceae	USA
		ZYMV	KC665634	C. pepo	Cucurbitaceae	USA
		ZYMV	KC665635	C. pepo	Cucurbitaceae	USA
		ZYMV	KF976712	C. pepo	Cucurbitaceae	Czechia
		ZYMV	KF976713	C. pepo	Cucurbitaceae	Slovakia
		ZYMV	KJ875864	C. pepo	Cucurbitaceae	USA
		ZYMV	KJ875865	C. pepo	Cucurbitaceae	USA
		ZYMV	KJ923767	C. pepo	Cucurbitaceae	USA
		ZYMV	KJ923768	C. pepo	Cucurbitaceae	USA
		ZYMV	KJ923769	C. pepo	Cucurbitaceae	USA
		ZYMV	KT598222	C. maxima	Cucurbitaceae	Argentina
ZYMV	crop	ZYMV	KT778297	C. anguria	Cucurbitaceae	India

		ZYMV	KU198853	C. pepo	Cucurbitaceae	Iran
		ZYMV	KY225542	pumpkin	Cucurbitaceae	Australia
		ZYMV	KY225543	pumpkin	Cucurbitaceae	Australia
		ZYMV	KY225544	pumpkin	Cucurbitaceae	East Timor
		ZYMV	KY225546	honey dew	Cucurbitaceae	Australia
		ZYMV	KY225547	rockmelon	Cucurbitaceae	Australia
		ZYMV	KY225548	honey dew	Cucurbitaceae	Australia
		ZYMV	KY225556	C. sativus	Cucurbitaceae	East Timor
		ZYMV	L29569	C. pepo	Cucurbitaceae	Reunion Island
		ZYMV	L31350	C. pepo	Cucurbitaceae	USA
		ZYMV	MF684760	C. pepo	Cucurbitaceae	Iran
		ZYMV	MG967620	G.max	Fabaceae	India
		ZYMV	MH427310	Apis mellifera-associated	Apidae	Australia
		ZYMV	MH700743	Cucumis sativus or C. pepo	Cucurbitaceae	Papua-NG
		ZYMV	MH700744	C. sativus or C. pepo	Cucurbitaceae	Papua-NG
		ZYMV	MH700745	C. sativus or C. pepo	Cucurbitaceae	Papua-NG
		ZYMV	MH700746	C. sativus or C. pepo	Cucurbitaceae	Papua-NG
		ZYMV	MH700747	C. sativus or C. pepo	Cucurbitaceae	Papua-NG
		ZYMV	MH700748	C. sativus or C. pepo	Cucurbitaceae	Papua-NG
		ZYMV	MH700749	C. sativus or C. pepo	Cucurbitaceae	Papua-NG
		ZYMV	MH700750	C. sativus or C. pepo	Cucurbitaceae	Papua-NG
		ZYMV	MH700751	C. sativus or C. pepo	Cucurbitaceae	Papua-NG
		ZYMV	MK124612	C. pepo	Cucurbitaceae	USA
		ZYMV	MN296124	C. pepo	Cucurbitaceae	China
		ZYMV	MN364667	Citrullus lanatus	Cucurbitaceae	Brazil
		ZYMV	MN598567	C. pepo	Cucurbitaceae	Australia
		ZYMV	MN598568	Cucurbita moschata	Cucurbitaceae	Australia
		ZYMV	MN598569	C. melo	Cucurbitaceae	Australia
		ZYMV	MN598571	C. pepo	Cucurbitaceae	Australia
		ZYMV	MN598572	C. lanatus	Cucurbitaceae	Australia
		ZYMV	MN598573	C. lanatus	Cucurbitaceae	Australia
		ZYMV	MN598574	C. lanatus	Cucurbitaceae	Australia
		ZYMV	MN598575	C. pepo	Cucurbitaceae	Australia
		ZYMV	MN598576	C. melo	Cucurbitaceae	Australia
		ZYMV	MN598577	C. pepo	Cucurbitaceae	Australia
		ZYMV	MN598579	C. melo	Cucurbitaceae	Australia
		ZYMV	MT497463	C. melo	Cucurbitaceae	Kenya
		ZYMV	MT882336	C. pepo	Cucurbitaceae	Iraq
		ZYMV	MW345248	C. sativus	Cucurbitaceae	Turkey
		ZYMV	MW345250	C. sativus	Cucurbitaceae	Turkey
		ZYMV	MW449264	C. pepo	Cucurbitaceae	France
		ZYMV	OK558793	C. pepo	Cucurbitaceae	Canada

		ZYMV	OK558794	C. pepo	Cucurbitaceae	Canada
		ZYMV	OL311706	C. melo	Cucurbitaceae	Israel
		ZYMV	OM471983	C. pepo	Cucurbitaceae	U.K.
		ZYMV	ON604832	C. pepo	Cucurbitaceae	Germany
		ZYMV	ON604841	C. pepo	Cucurbitaceae	Hungary
		ZYMV	ON604842	C. pepo	Cucurbitaceae	Reunion Is.
		ZYMV	OP357945	C. sativus	Cucurbitaceae	Sudan
		ZYMV	OP947584	C. sativus	Cucurbitaceae	Germany
		PWV	AB761400	P. edulis	Passifloraceae	Australia
		BSVA	JN052072	Chamaescilla corymbosa	Asphodelaceae	Australia
		HarMV	KJ152152	H. comptoniana	Fabaceae	Australia
		HarMV	KJ152153	Lupinus cosentinii	Fabaceae	Australia
		HarMV	KJ152154	H. comptoniana	Fabaceae	Australia
		HarMV	KJ152156	H. comptoniana	Fabaceae	Australia
		PWV	KX577780	P. caerulea	Passifloraceae	Australia
		PfVY	LC466655	P. edulis	Passifloraceae	Japan
		PaMNV	MW187786	Polygonatum kingianum	Asparagaceae	China
		PaVY	MZ190341	G. max	Fabaceae	Brazil
		WVMV	NC_007216	Wisteria spp.	Fabaceae	China
		PWV	NC_014790	P. caerulea	Passifloraceae	Australia
		HarMV	NC_015394	H. comptoniana	Fabaceae	Australia
		BSVA	NC_019415	Diuris sp.	Orchidaceae	Australia
	outgroup	PaMNV	NC_043532	Daiswa (Paris) polyphylla	Melanthiaceae	China

File S1. GenBank Accession numbers of the sequences compared.

AB100443
AB188115
AB188116
AB218280
AB246773
AB369278
AB369279
AB604610
AB690448
AB690449
AB690450
AB690451
AB690452
AB690453
AB690454
AB690455
AB761400
AF014811
AF241739
AJ307036
AJ310200
AJ312438
AJ312439
AJ316228
AJ316229
AJ507388
AJ515911
AJ619757
AJ628750
AM422386
AY112735
AY188994
AY216010
AY216987
AY278998
AY278999
AY279000
AY282577
AY294044
AY294045
AY575773
AY863025
AY968604
DQ124239
DQ399708
DQ666332
DQ674263
DQ821939
EF062582
EF062583
EF105298
EF105299
EU660578
EU660579
EU660580
EU660581
EU660582
EU660583
EU660584
EU660585
EU660586
EU660587
EU660588
EU660589
EU660590
EU761198
EU871724
EU871725
FJ376388
FJ548849
FJ640954
FJ640955
FJ640956
FJ640957
FJ640958
FJ640959
FJ640960
FJ640961
FJ640962
FJ640963
FJ640964
FJ640965
FJ640966
FJ640967
FJ640968
FJ640969
FJ640970
FJ640971
FJ640972
FJ640973
FJ640974
FJ640975
FJ640976
FJ640977
FJ640978

FJ640979	KC832502	KM051428	KT992082	KY225546
FJ640980	KC845321	KM051429	KT992083	KY225547
FJ640981	KC845322	KM051430	KT992084	KY225548
FJ640982	KF114860	KM051431	KT992085	KY225549
FJ807700	KF135488	KM076650	KT992086	KY225550
FJ807701	KF135489	KM597070	KT992087	KY225551
FJ823122	KF135490	KM597071	KT992088	KY225552
GQ219793	KF135491	KM597165	KT992089	KY225553
GU015011	KF274031	KM655833	KT992090	KY225554
GU214748	KF297335	KM979229	KT992091	KY225555
HG792063	KF439722	KP100058	KT992092	KY225556
HG792064	KF919297	KP114136	KT992093	KY242358
HM590054	KF919298	KP114137	KU198853	KY242359
HM590055	KF919299	KP164988	KU240094	KY249378
HQ161080	KF919300	KP710861	KU240095	KY614052
HQ166265	KF976712	KP710862	KU240096	KY659304
HQ166266	KF976713	KP710863	KU240097	KY659305
HQ229993	KF977830	KP710864	KU240098	KY659306
HQ229994	KF982784	KP710865	KU240099	KY769702
HQ229995	KJ152152	KP710866	KU240100	KY986929
HQ384216	KJ152153	KP710867	KU240101	L29569
HQ396725	KJ152154	KP710868	KU240102	L31350
HQ845735	KJ152156	KP710869	KU240103	LC037232
HQ845736	KJ152157	KP710870	KU240104	LC038070
HQ880242	KJ508092	KP710871	KU240105	LC038071
HQ880243	KJ645793	KP710872	KU240106	LC038072
JF273458	KJ786965	KP710873	KU240107	LC038073
JF273459	KJ807799	KP710874	KU240108	LC038077
JF273460	KJ807801	KP710875	KU240109	LC038078
JF273461	KJ807802	KP710876	KU240110	LC038079
JF273462	KJ807803	KP710877	KU246036	LC038080
JF273463	KJ807804	KP710878	KU528623	LC038081
JF273464	KJ807805	KP903372	KU896809	LC038082
JF273465	KJ807806	KR065437	KX096578	LC323107
JF273467	KJ807807	KT026108	KX249747	LC325839
JF273468	KJ807808	KT175568	KX302007	LC412927
JF273469	KJ807809	KT175569	KX421104	LC433691
JF833013	KJ807810	KT175570	KX499498	LC466655
JF833014	KJ807811	KT285170	KX505964	LC477217
JF833015	KJ807812	KT598222	KX512320	LC582403
JN052072	KJ807813	KT724930	KX577780	LC591946
JN183062	KJ807814	KT726938	KX664482	LC647195
JN416770	KJ807815	KT729506	KX664483	LC655955
JQ716413	KJ807816	KT778297	KX834319	LC656468
JX079685	KJ807817	KT992068	KX834320	LC683377
JX083210	KJ807818	KT992069	KX834321	LC723667
KC292915	KJ807819	KT992070	KX834322	LC729727
KC478389	KJ807820	KT992071	KX834323	MF040762
KC665627	KJ807821	KT992072	KX834324	MF072712
KC665628	KJ875864	KT992073	KX834325	MF072713
KC665629	KJ875865	KT992074	KX884565	MF072714
KC665630	KJ923767	KT992075	KX884570	MF078483
KC665631	KJ923768	KT992076	KX926428	MF179118
KC665632	KJ923769	KT992077	KY057338	MF179119
KC665633	KM023744	KT992078	KY225542	MF179120
KC665634	KM051425	KT992079	KY225543	MF405189
KC665635	KM051426	KT992080	KY225544	MF405190
KC832501	KM051427	KT992081	KY225545	MF405191

MF498886
MF498887
MF498888
MF498889
MF684760
MG194418
MG602227
MG602228
MG602229
MG602230
MG602231
MG602232
MG602233
MG602234
MG602235
MG640393
MG640394
MG640395
MG640396
MG640397
MG640398
MG640399
MG640400
MG640401
MG640402
MG640403
MG640404
MG640405
MG640406
MG640407
MG640408
MG640409
MG640410
MG640411
MG640412
MG640413
MG944249
MG967620
MG995842
MG995843
MH024838
MH024839
MH024840
MH024841
MH024842
MH024843
MH042024
MH042025
MH042026
MH169563
MH169564
MH169565
MH169566
MH169567
MH169568
MH220846
MH220847
MH270528
MH286883
MH427310
MH428831
MH469650
MH568695
MH628437
MH700743
MH700744
MH700745
MH700746
MH700747
MH700748
MH700749
MH700750
MH700751
MH744999
MH795801
MH844588
MH919384
MH919385
MH919386
MK033873
MK033874
MK069982
MK069983
MK069984
MK069985
MK069986
MK069987
MK069988
MK124612
MK217416
MK282414
MK340754
MK340755
MK350280
MK396065
MK427054
MK427057
MK449340
MK472693
MK906029
MK956829
MN124782
MN124783
MN296124
MN296125
MN316594
MN364667
MN399727
MN399738
MN514947
MN539670
MN598561
MN598562
MN598563
MN598564
MN598565
MN598566
MN598567
MN598568
MN598569
MN598571
MN598572
MN598573
MN598574
MN598575
MN598576
MN598577
MN598578
MN598579
MN598580
MN623289
MN623290
MN686524
MN686525
MN720007
MN722416
MN722417
MN722418
MN722419
MN723597
MN723598
MN729614
MN729616
MN734253
MN735448
MN735449
MN786956
MN854636
MN854637
MN854638
MN854639
MN854640
MN854641
MN854642
MN854643
MN854644
MN854645
MN854646
MN854647
MN854648
MN854649
MN854650
MN854651
MN914158
MN914159
MN914160
MN987554
MN987556
MN987557
MN987558
MN987559
MT067623
MT263075
MT364354
MT450870
MT497463
MT557572
MT603815
MT603816
MT603817
MT603818
MT603819
MT603826
MT603828
MT603829
MT603830
MT603831
MT603832
MT603833
MT603834
MT603835
MT603851
MT603852
MT648692
MT663309
MT672267
MT712111
MT741945
MT780536
MT780537
MT790744
MT882336
MT990976
MT990977
MW019501
MW019505
MW165064
MW187786
MW188031
MW197134
MW345248
MW345249
MW345250
MW345911
MW354946
MW354947
MW354948
MW354951
MW449260
MW449261
MW449262
MW449263
MW449264
MW464637
MW464638
MW483119
MW509731
MW534369
MW534370
MW582799
MW655827
MW675688
MW675689
MW701396
MW731696
MW731697
MW731698
MW731699
MW822167
MW822168
MW834586
MZ018226
MZ043618
MZ190341
MZ289075
MZ405649
MZ420677
MZ420678
MZ420679
MZ442684
MZ442685
MZ442686
MZ442687
MZ670770
NC_002634
NC_003224
NC_003397
NC_003537
NC_004013
NC_004047
NC_006262
NC_007216
NC_007728
NC_009741
NC_009742
NC_010954
NC_011560
NC_014790
NC_015394
NC_016159
NC_016441
NC_019415
NC_030236
NC_036802
NC_040650
NC_043532
NC_055470
NC_055601
OK050524
OK058515
OK094708
OK105105
OK156169
OK181786
OK558632
OK558633
OK558634
OK558658
OK558659
OK558660

OK558793
OK558794
OK558795
OK625818
OL311706
OL472139
OL584353
OL753658
OL871237
OM108477
OM128429
OM471983
OM621805
OM746964
OM948812
OM948813
OM948814
OM948815
OM948816
OM948817
OM948818
OM948820
OM948821
OM948823
OM948824
OM948825
OM948826
OM948827
OM948828
OM948830
OM948831
OM948832
OM948833
ON013906
ON086743
ON604832
ON604841
ON604842
ON641738
ON843744
ON843745
ON843746
ON843747
ON843748
ON843749
ON843750
ON924225
OP357945
OP380926
OP564897
OP828732
OP947577
OP947584
S42280
U05771
U34972

Figure S1. Branching pattern of the neighbor-joining phylogeny of the principal genomic ORFs of 731 BCMV subgroup viruses. The clusters of each of the nine major 'crop' viruses are shown**.**

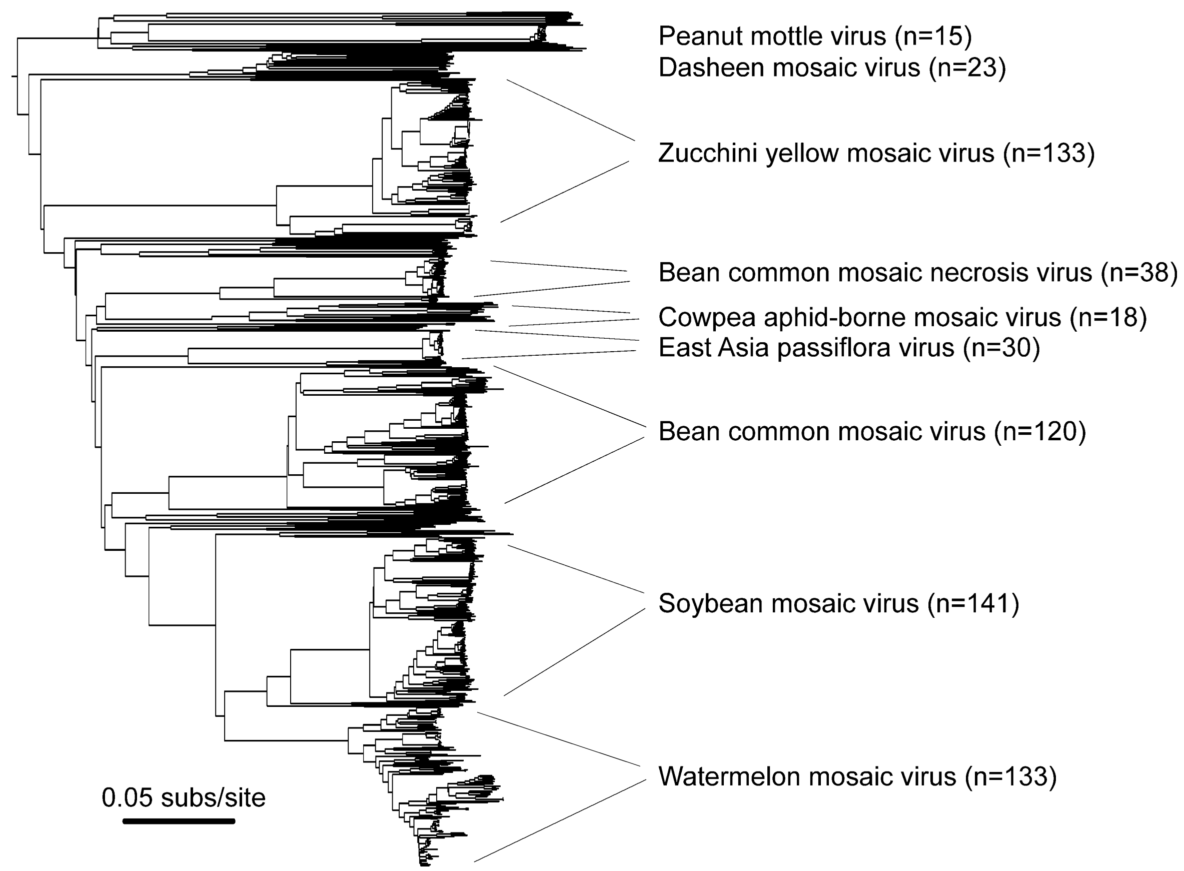

Figure S2. Vital statistics of the 731 sequence alignment (including indels)

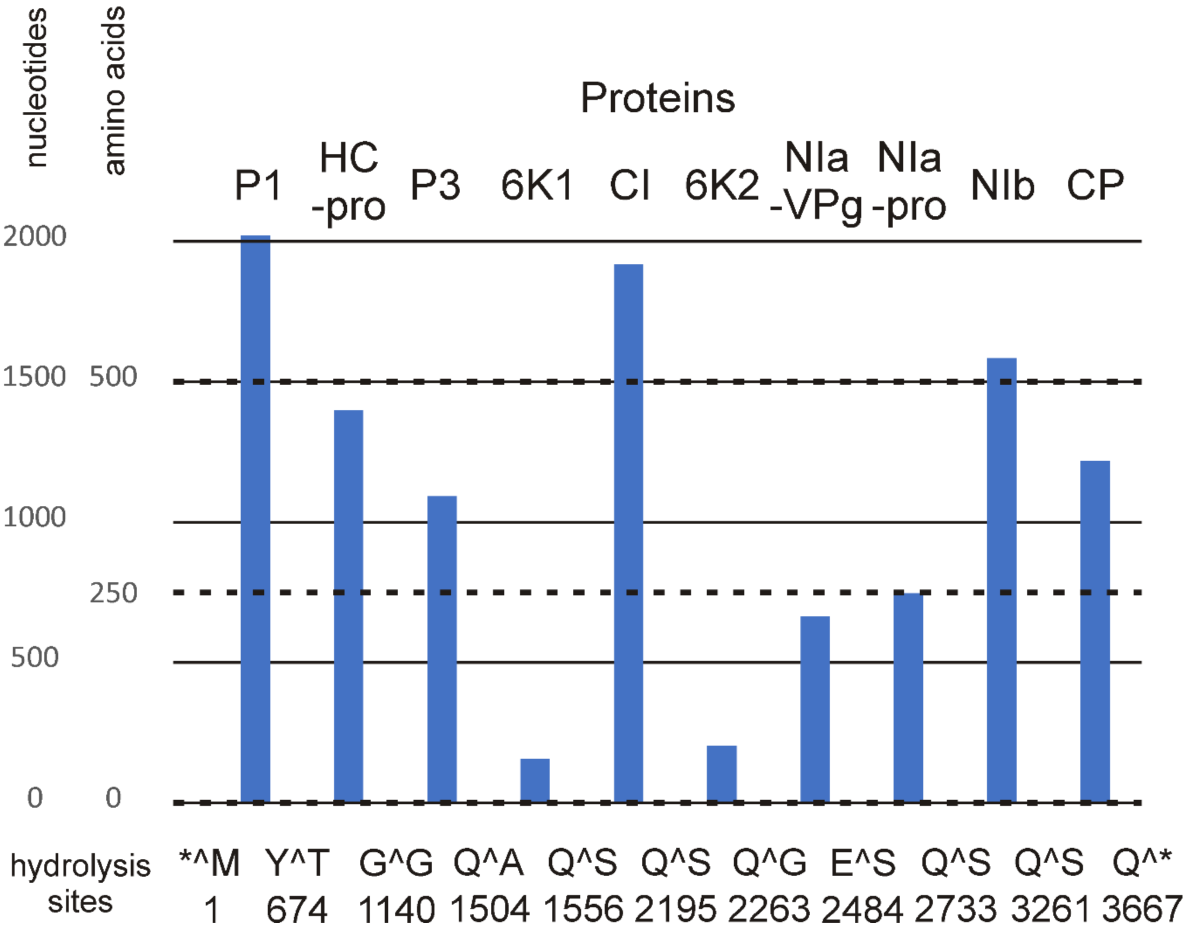

Table S2. Results of FUBAR searches for negatively and positively selected codons in the main ORFs of seven crop viruses and their corresponding outgroup viruses.

Virus	Number of sequences	Number of negatively selected codons	Number of positively selected codons	Positively selected codons*	Genes containing positively selected codons
BCMNV-all	32	3324	4	**1316, 1326, 1336,1340**	P3, P3, P3, P3, P3
BCMNV-crop	17	1300	0	-	-
BCMNV-outgroup	15	3299	3	**1316, 1326, 1336**	P3, P3, P3
BCMV-all	60	3332	8	**1316, 1326, 1336, 1340, 1343, 1352, 1355,** 1547	P3, P3, P3, P3, P3, P3, P3, 6K1
BCMV-crop	45	2238	0	-	-
BCMV-outgroup	15	3373	3	**1326, 1336, 1355**,	P3, P3, P3
CpAbMV-all	23	3342	2	**1336, 1340**	P3, P3
CpAbMV-crop	8	2852	1	2392	NIa-VPg
CpAbMV-outgroup	15	2963	1	1087	HC-pro
DashMV-all	26	3422	3	**1316, 1336, 1347**	P3, P3, P3
DashMV-crop	11	3178	2	509, 2780	P1, NIb
DashMV-outgroup	15	3329	2	**1316, 1355**	P3, P3
EAPV-all	43	3351	4	**1316, 1326, 1336, 1355**	P3, P3, P3, P3
EAPV-crop	28	1637	2	599, 781	P1, HC-Pro
EAPV-outgroup	15	3312	1	**1316**	P3
PeMoV-all	30	3393	1	614	P1
PeMoV-crop	15	866	4	614, 740, **1409,** 2261	P1, HC-pro, P3, 6K2
PeMoV-outgroup	15	3347	1	**1351**	P3
ZYMV-all	90	3411	1	**1316**	P3
ZYMV-crop	75	2982	1	462	P1
ZYMV-outgroup	15	3312	2	**1316, 1355**	P3, P3

* Codons numbered as in the 731 sequence alignment (see Methods). Codons found to be positively selected in >5 analyses are bolded.

Table S3. Results of FUBAR searches for negatively and positively selected codons in the P3 genes of seven crop viruses and their corresponding outgroup viruses.

Source of ORFs	Number of ORFs	Number of negatively selected codons	Number of positively selected codons	Positively selected codons*
BCMNV-all	32	247	4	**1316**, **1326**, **1336**, **1340**
BCMNV-crop	17	41	0	-
BCMNV-outgroup	15	234	3	**1316**, **1326**, **1336**
BCMV-all	60	262	9	**1316**, 1321, **1326**, **1336**, **1340**, 1346, **1347**, 1352, 1355
BCMV-crop	45	137	2	1147, 1462
BCMV-outgroup	15	241	6	**1326**, **1336**, **1340**, 1343, **1347**, 1355
CpAbMV-all	23	263	6	1312, **1326**, **1336**, **1340**, **1347**, 1354
CpAbMV-crop	8	192	1	1485
CpAbMV-outgroup	15	229	3	**1326**, **1336**, 1340
DashMV-all	26	267	6	**1316**, **1336**, 1344, **1347**, 1349, 1368
DashMV-crop	11	232	3	1335, 1382, 1488
DashMV-outgroup	15	233	4	**1316**, **1326**, 1343, **1347**
EAPV-all	43	264	8	**1316**, **1326**, **1336**, **1340**, 1343, **1347**, 1348, 1355
EAPV-crop	28	100	1	1348
EAPV-outgroup	15	233	4	**1316**, **1326**, 1343, **1347**
PeMoV-all	30	244	5	**1326**, **1340**, **1347**, 1355, 1359
PeMoV-crop	15	22	0	-
PeMoV-outgroup	15	233	4	**1316**, **1326**, 1343, **1347**
ZYMV-all	90	263	4	**1316**, **1326**, 1344, 1351
ZYMV-crop	75	203	2	1332, 1351
ZYMV-outgroup	15	232	2	**1316**, 1355

* Codons numbered as in the 731 sequence alignment (see Methods). Codons found to be positively selected in >5 analyses are bolded.
